# Health performance assessment modeling and its application to compact medical communities in China

**DOI:** 10.1002/hcs2.105

**Published:** 2024-07-23

**Authors:** Miao Yu, Zhongmou Huang, Dan Zhang, Yansui Yang, Ching‐Wen Chien, Hongwu Tuo

**Affiliations:** ^1^ Institute for Hospital Management Tsinghua University Beijing China; ^2^ Institute for Hospital Management, Shenzhen International Graduate School Tsinghua University Shenzhen China; ^3^ Guangzhou College of Technology and Business Guangzhou China

**Keywords:** compact medical community, health performance assessment, medical insurance

## Abstract

Some regions in China have already implemented capitation payment or capitation budget management for medical insurance funds. However, there remains a shortage of adequate tools and methodologies to accurately quantify differences in population health risks. Therefore, this paper constructs a health performance assessment model that comprises four steps. The first step is to categorize all participants into health risk groups based on whether they have contracted with a family doctor, their age, sex, and the type of consultation. The second step is to categorize health risk groups based on differences in healthcare resource utilization. The third step is to analyze health performance by examining healthcare resource utilization year over year. The fourth step is to apply the assessment results to assist local finance bureaus and medical insurance bureaus in developing incentive schemes. According to cost weights, the health risk groups are split into six classes: insured residents without health care visits, healthy insured person, slightly ill insured patients, ill insured patients, more seriously disease patients, and severely ill insured patients. We evaluate one compact medical community's health performance by examining changes in the proportion of resource usage group size and expense. From 2019 to 2021, both the proportion of patients with severe and ultra‐severe diseases and the proportion of costs in the sample increased, according to changes in resource utilization levels. This result indicates that the population's overall health has not improved and that the compact medical community is still primarily focused on treating diseases, with poor implementation of health maintenance measures and minimal improvement in health performance.

## Graphical abstract

The health performance assessment model comprises four steps: 1. Grouping by health risk; 2. Grouping by resource utilization; 3. Assessing health performance; 4. Incentive schemes. This model could help medical insurance funds allocate capitation budgets for various populations in accordance with variations in health risks and provide incentives to compact medical communities which are successful in promoting health.

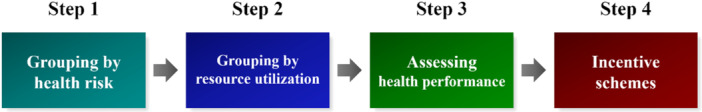

**Abbreviations**
ACG: Adjusted Clinical Group
HRG: Health Risk Group

## 1 INTRODUCTION

China is striving to adopt a population‐health‐centered healthcare delivery system. Such a system must include established methods of measuring and paying for health improvement. Thus, the issue of how to adopt a healthcare delivery model focused on population health affects payments to China's compact medical communities in two ways: by influencing both the financial subsidies offered by the Chinese government and the payments made by medical insurance funds. In some Chinese cities, medical insurance funds have already implemented capitation payment or capitation budget management, but there is a shortage of tools and methodologies to quantify differences in population health risks and to apply differential budgets based on those disparities. As a result, the population's health performance in compact medical communities is not yet being improved by the current medical insurance payment mechanisms.

The objective of matching patients' use of clinical healthcare resources with their clinical status is to distinguish health performance assessment from general health evaluation and healthcare service satisfaction assessment. Medical facilities can be encouraged to manage residents' health by conducting health assessments when people sign up at family doctors' offices. They can then use the balance of the medical insurance fund for performance rewards after the assessments. The resulting “pay‐for‐value” system, whereby payment is based on health outcomes, would be supported by health performance assessments conducted by medical insurance funds. Such a system would encourage medical insurance funds to shift away from making payments to treat diseases, instead using payments to maintain health [1].

Research on cost‐ and performance‐based reimbursement models for healthcare delivery has already begun in some countries. The Johns Hopkins Adjusted Clinical Group (ACG) conducted a representative study [2] and developed a method of classifying patients based on clinical information. The method divides people into groups according to the severity of the diseases they have been diagnosed with over time. Based on the quantity of resources devoted to patient comorbidities, the researchers aimed to assess the severity of disease in a patient population [3]. The ACG system is effective at analyzing the connection between disease burden or morbidity and the use of healthcare services, and it can serve as a useful model for reforming the entire system of medical insurance payment in China's compact medical communities. However, the ACG system in the United States, while providing valuable insights for the study of global payment in China, cannot be directly applied due to significant differences in the primary healthcare service system, patient consultation patterns, the diagnostic and treatment practices of primary care physicians, and the classification of International Classification of Diseases. As a result, it is not yet possible to fully replicate and implement the ACG system in China [4].

To construct a health performance assessment model and test it using data from a sample of districts in China, this research synthesized patient grouping and global health performance assessment approaches and integrated them with the features of China's healthcare delivery system. We built an operationalized health risk grouping model and health performance assessment model for China's medical insurance. The model presented in this article could help medical insurance funds allocate capitation budgets for various populations in accordance with variations in health risks. It also has the potential to provide incentives to compact medical communities that are successful in promoting health. The findings of this study will advance value‐based payment reform and health performance assessment, and it will encourage compact medical communities to actively maintain the health of their residents.

## 2 METHODS

### 2.1 Framework

**Figure 1:**
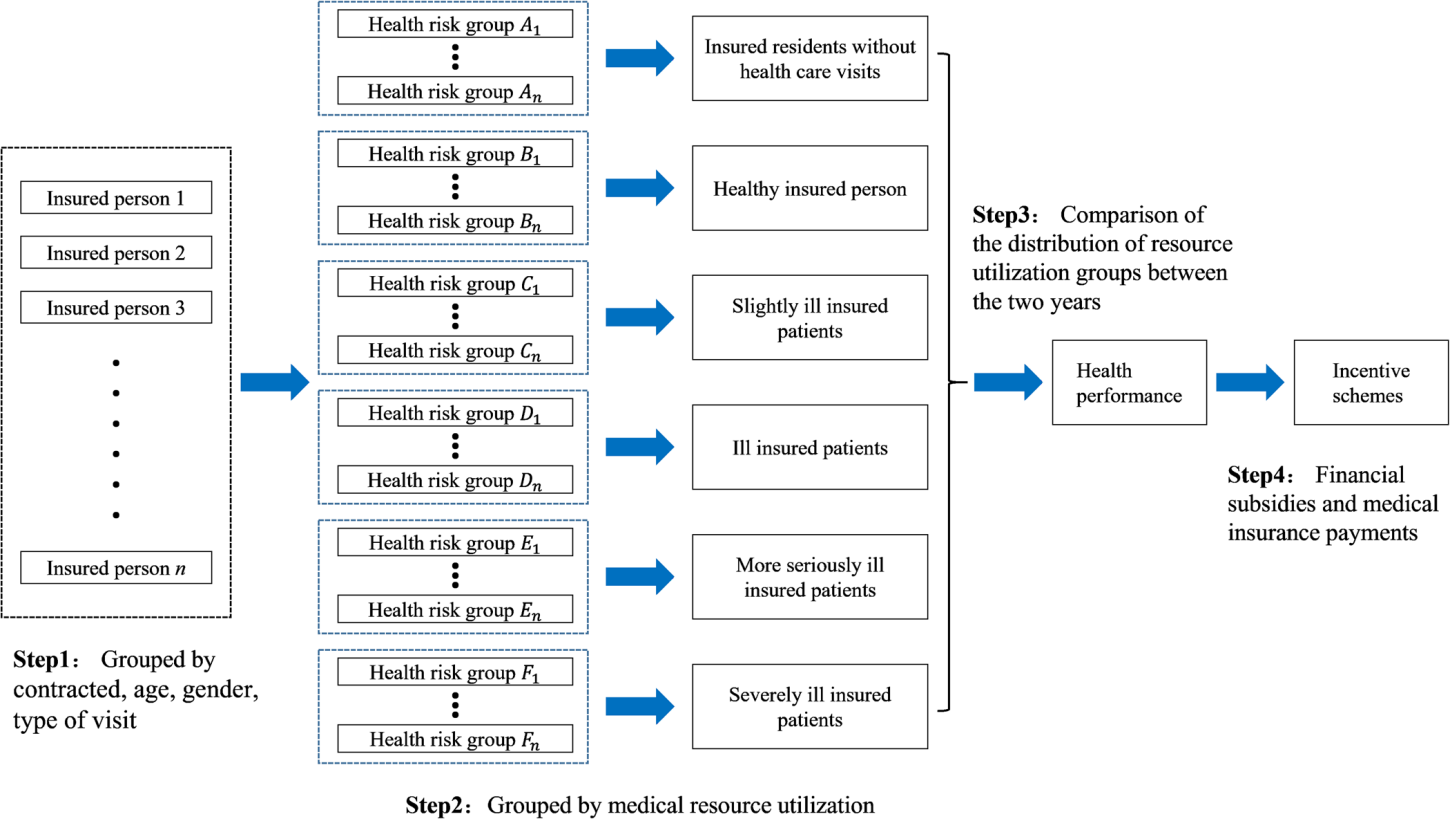
Health Performance Assessment Framework.

The four steps of the health performance assessment are as follows (Figure 1). The first step is to divide all participants into health risk groups based on their age, sex, and the type of visit (general outpatient, chronic disease outpatient, special disease outpatient, and hospitalization), as well as whether they have a family doctor. The second step is to subdivide the health risk groups into six resource utilization groups, based on the cost distribution of resource utilization. These six groups are insured individuals without health care visits, healthy insured person, slightly ill insured patients, ill insured patients, more seriously disease patients, and severely ill insured patients. The third step is to compare health performance among the resource utilization groups between the 2 years of the study. The fourth step is to apply the assessment results to assist local medical insurance bureaus in developing incentive schemes.

### 2.2 Health performance assessment procedure

#### 2.2.1 Step 1: Grouping by health risk

Following existing literature, we grouped all participants based on four indicators: whether or not they had a contract with a family doctor, age, sex, and type of visit. There are six age groups: early childhood (0–6 years old), junior (7–17 years old), youth (18–45 years old), middle age (46–59 years old), senior (60–79 years old), and high age (80 years old and above). Sex is divided into male and female. Visit types are defined by consultation category and number of visits. The four primary categories of consultation are general outpatient, outpatient chronic disease, outpatient special disease (individuals with prolonged illnesses and significant medical costs that are better suited for outpatient care, which is more cost‐effective and convenient than hospitalization), and hospitalization. In China, patients affiliated with chronic and specialty diseases must undergo a qualification process facilitated by the medical insurance department. Reimbursement for related medical expenses is contingent upon approval for the health insurance authority. The criteria for identifying these special and chronic diseases are established by the local medical insurance department and are subject to their evaluation and determination. The number of visits is categorized into five levels: 0, 1, 2–3, 4–5, and 6 and above. Through five steps (Supporting Information S1: Appendix A), we grouped participants into the 214 health risk groups.

#### 2.2.2 Step 2: Grouping by resource utilization

After completing the health risk grouping, we use weighted medical expenses to measure the consumption of medical resources, dividing participants into six groups according to variations in the consumption and utilization of health care resources among the health risk groups. Group 0 consists of unattended enrolled participants, referring to those who are enrolled but without recorded health facility visits. This group is characterized by no consumption of health care resources, and thus the weight of medical expenses is set at 0. Group 1 consists of healthy insured participants who, despite their health facility visits, consume few healthcare resources. The weight of medical expenses is set at 0–0.2. Group 2 consists of slightly ill insured participants, who exhibit both visit behavior and low health care resource consumption, so the weight of medical expenses is set at 0.2–1. Group 3 contains ill insured participants, defined as patients with visit behavior and higher‐than‐average consumption of health care resources. The weight of medical expenses is set at 1–4. Group 4 consists of more seriously ill participants, who have a high visit rate and high resource consumption. Medical expenses are weighted at 4–8. Group 5 is made up of insured participants who have more serious diseases and use more medical resources than other groups, and the weight of cost is set to be higher than 8.

#### 2.2.3 Step 3: Using resource utilization to assess health performance

We counted the number of insured participants and the cost of each resource utilization group and analyzed the trend to assess the correlation between health status and the medical resource utilization. This correlation was then used to evaluate the health performance of the compact medical community. If the proportion of participants in Groups 0 and 1 rises and that of participants in Groups 4 and 5 decreases, we can conclude that the healthy population is increasing and the overall health status of the region is improving. If the proportion of expenses in Groups 0 and 1 increases and that of expenses in Groups 4 and 5 decreases, we can conclude that medical expenses are more used for health management and prevention, rather than disease treatment, and the effectiveness of health maintenance is improving.

#### 2.2.4 Step 4: Developing incentive schemes

We recommend that China implement generous economic incentives for integrated medical communities with high health performance and significant improvements. An effective health performance assessment system will help enhance quality and safety of medical care, as well as the health of the residents of the entire integrated medical community.

## 3 CASE STUDY

### 3.1 Study population

Our sample of insured participants was drawn from Gongyi City, which is a county‐level city in Henan Province. We utilized data obtained from the Gongyi Health Insurance Bureau, which collects medical visit data for all residents from the medical insurance system in Gongyi City. Our sample consisted of data from 2019 to 2021. This data comprises flow data for inpatient care, general outpatient care, and outpatient care for both chronic and special diseases. The medical institutions visited include all types, such as hospitals, community health service centers, and township health centers.

### 3.2 Patient grouping

#### 3.2.1 Health risk groups

Group 1 refers to patients who do not attend the clinic. The number of non‐attending patients is equal to the number of insured residents minus insured patients attending the clinic. The mean cost of treatment for Group 1 is zero. Groups 2–214 are the groups of patients attending the clinic. The group with special diseases includes patients with outpatient special diseases but without outpatient chronic diseases. The group with both special and chronic diseases includes outpatients. Although the number of young children with chronic diseases is small, they are grouped separately owing to the distinctive characteristics of this patient group. The remaining participants are grouped according to the health risk grouping model described above. The mean cost of each group is computed by dividing the aggregate cost of the group by the total number of individuals.

#### 3.2.2 Resource utilization groups

According to the weighting rules, this paper divided 213 health risk groups into 6 resource utilization groups. The number of health risk groups was the highest in the group of severely ill insured patients: 66, 77, and 78 in 2019, 2020, and 2021, respectively. The lowest number of health risk groups was 10, 14, and 19, in the slightly ill insured patients' group, respectively.

### 3.3 Health performance assessment

Trends in the percentage of each resource utilization group from 2019 to 2021 showed that the percentage of the group of unattended enrolled residents increased in 2020 and decreased slightly in 2021, but increased in both 2020 and 2021 compared with 2019. The percentage of healthy insured patients decreased from 39.01% in 2019 to 34.28% in 2020 and 31.43% in 2021. The percentage of slightly ill insured patients increased from 17.59% in 2019 to 17.98% in 2020 and 22.51% in 2021. The percentage of ill insured patients decreased from 5.87% in 2019 to 3.39% in 2020 but rose to 3.62% in 2021. The percentage of more seriously ill insured patients increased from 5.49% in 2019 to 6% in 2020 and fell to 5.64% in 2021. The percentage of severely ill insured patients trended upward, from 2.61% in 2019 to 3.5% in 2020 and 4.01% in 2021 (Table 1).

**Table 1:** Distribution of participants by resource utilization group, 2019–2021.

Resource utilization group	2019		2020		2021	
	Number of people	Percentage (%)	Number of people	Percentage (%)	Number of people	Percentage (%)
0	194,742	29.45	231,244	34.86	218,850	32.80
1	257,985	39.01	227,382	34.28	209,739	31.43
2	116,307	17.59	119,259	17.98	150,185	22.51
3	38,822	5.87	22,458	3.39	24,126	3.62
4	36,279	5.49	39,806	6.00	37,636	5.64
5	17,230	2.61	23,218	3.50	26,743	4.01
Total	661,365	100.00	663,367	100.00	667,279	100.00

### 3.4 Incentive schemes

In Gongyi City, there is only one integrated healthcare community, led by Gongyi General Hospital, and the budget for each fund participant is allocated to Gongyi General Hospital. In areas with multiple integrated healthcare communities, the budget (Supporting Information S1: Appendix B) for each participant is allocated to the healthcare community to which that participant belongs, and the total budget of the medical insurance fund is obtained by adding up the budgets of all the participants.

When paying for healthcare services, the budget of the contracted participant and the actual expenses incurred will be compared, the excess will not be compensated, and the balance will be retained. In this way, the health care community is encouraged to actively control the growth of health care costs and maintain the health of patients.

## 4 CONCLUSION

In this article, we validated the feasibility of a health performance assessment model using data from a sample of districts. We grouped insured residents by their health risk status and calculated the resource utilization level for each group. Resource utilization levels indicated a higher percentage of the number of patients in the more seriously ill and severely ill patient groups in 2019–2021. This suggests that overall population health had not improved and the healthcare community prioritizes disease treatment over health maintenance, with little improvement in health performance in the sample area.

The contribution of this paper is to establish a new grouping model of insurance fund participants based on health‐centered principles, and to measure the health performance of insured populations using changes in resource utilization level as a proxy. This paper helps address the problem of how to measure health performance using data generated by the payment mechanism of integrated medical services.

The results reported in this study should be considered in light of some limitations. First, the health risk grouping is measured only in terms of the type and number of healthcare services utilized, and lacks a measure of the severity of the illnesses suffered by insured patients. Second, the resource utilization levels are divided using cost weights. This results in groups that are relatively homogeneous and do not adequately reflect the differences in the consumption of medical resources. Finally, the evaluation of health performance uses changes in the number and cost of resource utilization groups, which is a result‐level indicator. There is a lack of measurement indicators for the performance of health management and medical services. This may be problematic if the health performance evaluation does not fully reflect the performance of the compact medical communities.

## AUTHOR CONTRIBUTIONS

**Miao Yu**: Data curation; methodology; writing—original draft, review, and editing. **Zhongmou Huang**: Data curation; writing—original draft, review, and editing. **Dan Zhang**: Conceptualization; methodology. **Yansui Yang**: Conceptualization; methodology; writing—review. **Ching‐Wen Chien**: Conceptualization; methodology. **Hongwu Tuo**: Data curation; methodology; formal analysis; writing—original draft and review.

## CONFLICT OF INTEREST STATEMENT

Professor Yansui Yang is the member of the *Health Care Science* Editorial Board. To minimize bias, she was excluded from all editorial decision‐making related to the acceptance of this article for publication. The remaining authors declare no conflict of interest.

## ETHICS STATEMENT

Not applicable.

## INFORMED CONSENT

Not applicable.

## Supporting information

Supporting information.

## Data Availability

No data is available.
