# Supplementary material for: Health performance assessment modeling and its application to compact medical communities in China
Source: Health Care Sci. 2024 Jul 23;3(4):232–7. doi: 10.1002/hcs2.105 (PMC11362657; doi:10.1002/hcs2.105)
Supplement: Supplementary file 1 — Supporting information. [file HCS2-3-232-s001.docx]

**SUPPLEMENTARY** **MATERIAL**

**Appendix A**

in the following order: disease status, age, sex, type of medical visit, and number of medical consultations. In the first step, we created two groups, depending on whether patients had a special disease; ‘Group S-1’ represents those who do and ‘Group S-2’ represents those who do not. In the second step, these groups were further divided according to whether they suffer from a chronic disease; ‘Group SC-1’ and ‘Group SC-3’ represent those who do, and ‘Group SC-2’ and ‘Group SC-4’ represent those who do not. In the third step, the groups were further subdivided into ‘Group SCAS-1’, ‘Group SCAS-2’, ‘Group SCAS-3’, ..., ‘Group SCAS-48’ based on the six age groups (Figure 2). In the fourth step, the groups were further divided into the five health risk groups described above. In the fifth step, the groups were further divided based on the number of visits, again using the five categories described above. To create the final health risk groups, groups with fewer members were eventually combined into similar groups.


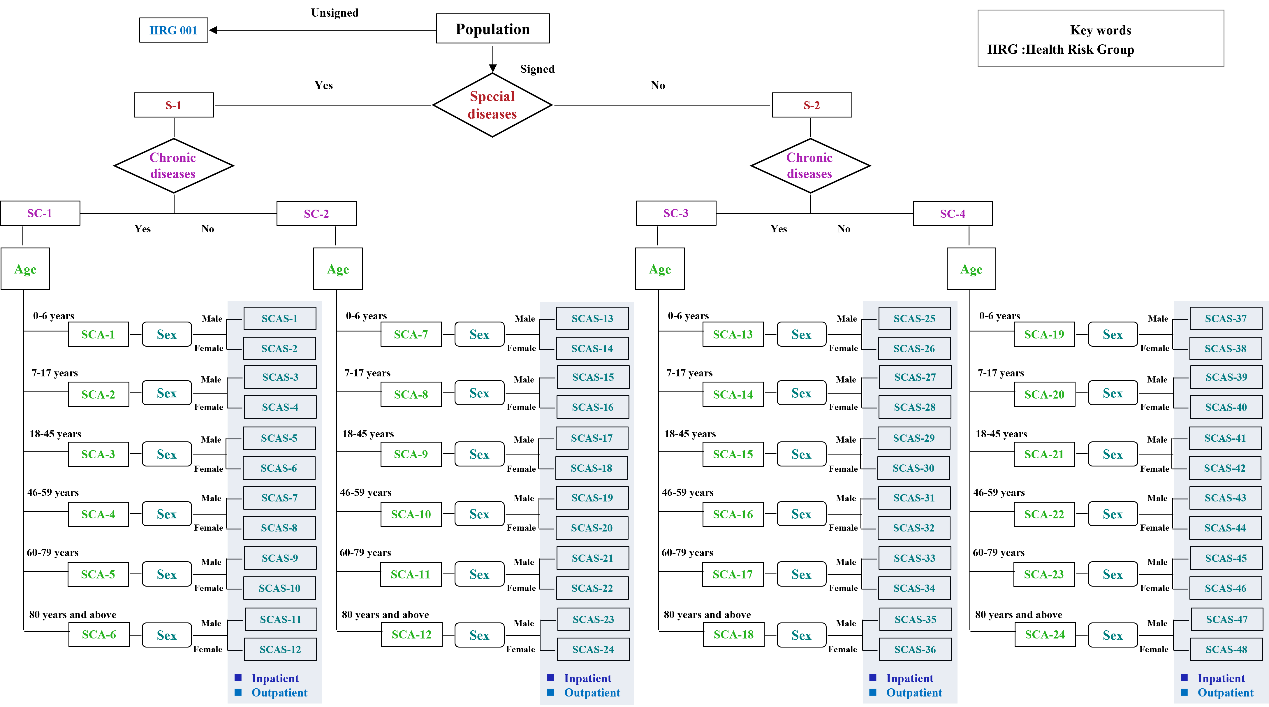


**APPENDIX FIGURE 1** Health Risk Grouping Model

Abbreviation: HRG, Health Risk Group.

**Appendix B**

The budget of the medical insurance coordination fund is allocated to each participant according to the weight of the health risk group. First, we used cost weights to modify the fund’s capitation budget for participants. The average capitation budget allocated to each fund participant can be calculated by dividing the total budget of the fund by the sum of the product of the number of individuals in each risk group and their corresponding weights. Second, the average capitation budget for each health risk group is obtained by multiplying the average capitation budget for all enrollees with the cost weights for each health risk group. Third, the average capitation budget for each health risk group is multiplied by the number of people in the group to obtain the total budget for that health risk group. Fourth, summing the total budget of each health risk group gives the total budget for the medical insurance fund of the integrated healthcare community. The per capita budget of the insured person is equal to the average capitation budget of the health risk group in which the insured person belongs.
